# Sand Fly Salivary Proteins Induce Strong Cellular Immunity in a Natural Reservoir of Visceral Leishmaniasis with Adverse Consequences for *Leishmania*

**DOI:** 10.1371/journal.ppat.1000441

**Published:** 2009-05-22

**Authors:** Nicolas Collin, Regis Gomes, Clarissa Teixeira, Lily Cheng, Andre Laughinghouse, Jerrold M. Ward, Dia-Eldin Elnaiem, Laurent Fischer, Jesus G. Valenzuela, Shaden Kamhawi

**Affiliations:** 1 Vector Molecular Biology Unit, Laboratory of Malaria and Vector Research, National Institute of Allergy and Infectious Diseases, National Institutes of Health, Bethesda, Maryland, United States of America; 2 Comparative Medicine Branch, National Institute of Allergy and Infectious Diseases, National Institutes of Health, Bethesda, Maryland, United States of America; 3 Laboratory of Malaria and Vector Research, National Institute of Allergy and Infectious Diseases, National Institutes of Health, Bethesda, Maryland, United States of America; 4 Merial S.A.S, R&D, Laboratoire de Lyon Gerland, Lyon, France; Stanford University, United States of America

## Abstract

Immunity to a sand fly salivary protein protects against visceral leishmaniasis (VL) in hamsters. This protection was associated with the development of cellular immunity in the form of a delayed-type hypersensitivity response and the presence of IFN-γ at the site of sand fly bites. To date, there are no data available regarding the cellular immune response to sand fly saliva in dogs, the main reservoirs of VL in Latin America, and its role in protection from this fatal disease. Two of 35 salivary proteins from the vector sand fly *Lutzomyia longipalpis*, identified using a novel approach termed reverse antigen screening, elicited strong cellular immunity in dogs. Immunization with either molecule induced high IgG_2_ antibody levels and significant IFN-γ production following *in vitro* stimulation of PBMC with salivary gland homogenate (SGH). Upon challenge with uninfected or infected flies, immunized dogs developed a cellular response at the bite site characterized by lymphocytic infiltration and IFN-γ and IL-12 expression. Additionally, SGH-stimulated lymphocytes from immunized dogs efficiently killed *Leishmania infantum chagasi* within autologous macrophages. Certain sand fly salivary proteins are potent immunogens obligatorily co-deposited with *Leishmania* parasites during transmission. Their inclusion in an anti-*Leishmania* vaccine would exploit anti-saliva immunity following an infective sand fly bite and set the stage for a protective anti-*Leishmania* immune response.

## Author Summary

Leishmaniasis is a neglected infectious disease with a global distribution encompassing 88 countries, 350 million people at risk, and an annual incidence of 2 million cases. Leishmaniasis is a vector-borne disease transmitted by sand fly bites where parasites are co-deposited with saliva into the wound. Our group has demonstrated that distinct molecules in the saliva of various sand fly species drive an immune response that protects experimental rodent models from self-healing cutaneous and fatal visceral leishmaniasis. Here we show for the first time that dogs, natural reservoirs of visceral leishmaniasis, develop a strong immune response to two salivary proteins from the natural vector sand fly. Blood from immunized dogs contained immune cells that produced molecules (IFN-γ) typically associated with protection from *Leishmania* parasites. This response efficiently recruited appropriate immune cells to the site of sand fly bites in the skin and had an adverse effect on *Leishmania* parasites in an experimental assay. These findings suggest that inclusion of these salivary molecules in anti-*Leishmania* canine vaccines would enhance their efficiency in protecting dogs from visceral leishmaniasis. A successful anti-*Leishmania* canine vaccine would not only protect dogs from a fatal disease but could have a considerable effect on reducing human infections.

## Introduction

Leishmaniasis is a vector-borne neglected disease transmitted exclusively by the bite of infected phlebotomine sand flies. An estimated 350 million people are at risk for leishmaniasis with an annual incidence of 2 million cases and a loss of 2,357,000 disability-adjusted life years [1],[2],[3]. Leishmaniasis presents with multiple clinical manifestations including cutaneous, mucocutaneous, diffuse and visceral (VL) infections. The latter is responsible for 59,000 deaths a year, a parasitic disease statistic surpassed only by malaria [4]. There are two types of VL, anthroponotic and zoonotic. Zoonotic VL (ZVL) is wide spread and occurs in Latin America, Northern Africa, Southern Europe and areas of the Middle East and Asia [5],[6],[7]. The dog is considered the main reservoir of ZVL [5],[8]. Indeed, there is a clear association between a high rate of infection in dogs and an increased risk of human disease [3]. An anti-*Leishmania* canine vaccine would not only protect dogs from a fatal disease but could have a considerable effect on reducing human infections.

Understandably, the search for vaccine candidates for leishmaniasis has focused on *Leishmania* antigens [9]. Several promising first, second and third generation vaccine candidates produced variable levels of protection in animal models [9]. Still there are no available human vaccines for any form of leishmaniasis and LEISHMUNE, a canine vaccine based on a *Leishmania infantum chagasi* fucose-mannose-ligand glycoprotein fraction [10], is only licensed in Brazil [11]. Although LEISHMUNE has demonstrated some efficacy against canine visceral leishmaniasis (CVL) it has limitations that include safety issues and the difficulty to serologically distinguish asymptomatic from vaccinated dogs [11],[12].

Sand fly salivary proteins are inoculated at the site of parasite deposition during transmission by infective sand fly bites. Thus, immunogenic salivary proteins that influence the immune status of the host can potentially have consequences on the outcome of leishmaniasis. This hypothesis has been corroborated in rodent models where immunization with sand fly saliva or a distinct salivary protein conferred protection against both cutaneous and visceral leishmaniases [13],[14],[15],[16],[17]. This protection has been correlated with a Th1 response against salivary antigens characterized by the presence of IFN-γ at the bite site [14],[17].

The above puts forward a solid argument for the use of salivary gland proteins of appropriate vector sand fly species to improve the efficacy and immunogenicity of *Leishmania*-based vaccine candidates. In this study, immunization of dogs with two novel salivary proteins from *Lutzomyia longipalpis*, the only established vector of *L. i. chagasi* in Latin America, resulted in a strong systemic and local Th1 cell-mediated immunity that was efficiently recalled by sand fly bites and adversely affected parasite survival *in vitro.* To our knowledge, this is the first demonstration that specific immunity to a salivary protein can be elicited in a natural host of the *Leishmania* parasite and an endorsement for the use of salivary proteins, neglected thus far, as novel antigens in anti-*Leishmania* vaccines.

## Results

### Bites of *Lutzomyia longipalpis* sand flies induce a strong delayed type hypersensitivity response in dogs

**Figure 1:**
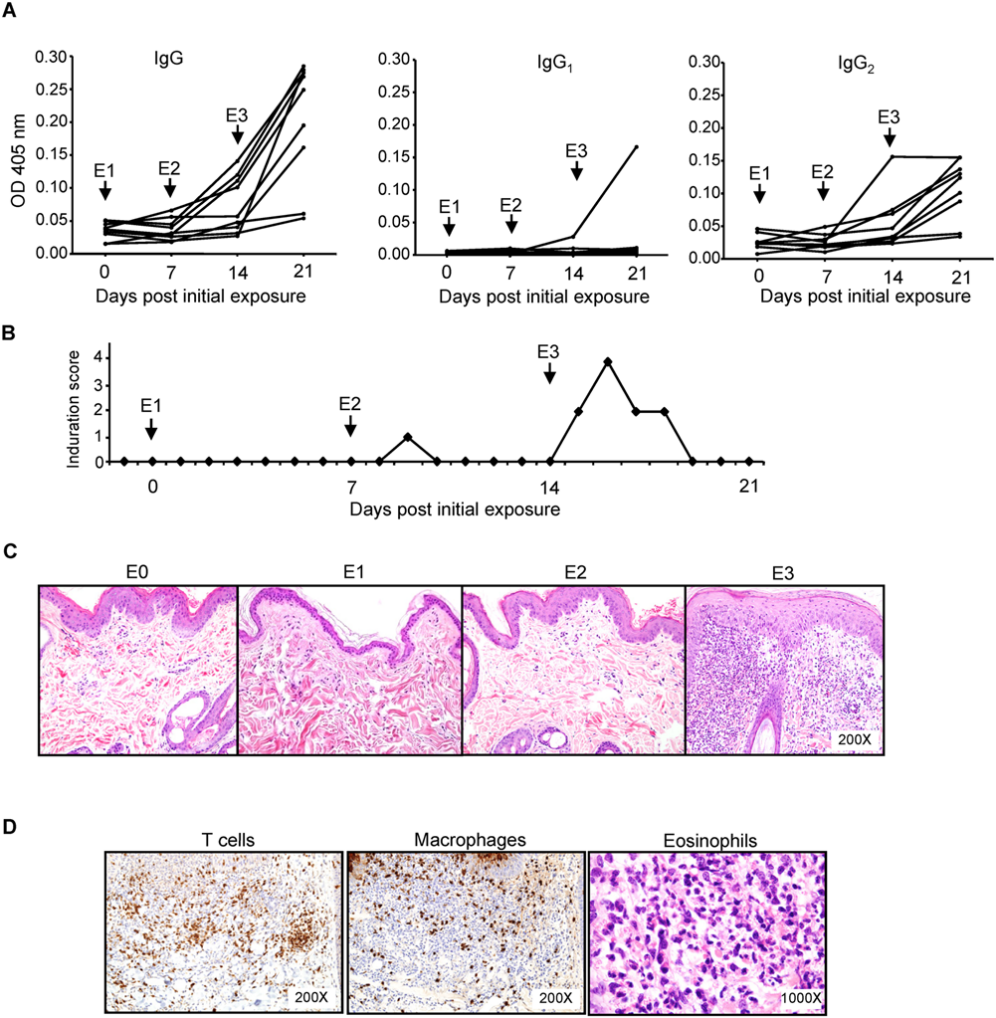
Dogs develop a strong humoral and cellular immune response to bites of *Lu. longipalpis* sand flies. Dogs (n = 9) were exposed for 10 min to bites of 20 sand flies three times at one week intervals (first exposure, E1; second exposure, E2; third exposure, E3). (A) Weekly measurement of IgG, IgG_1_ and IgG_2_ antibody levels in dogs exposed to sand flies. (B) Induration score in a representative dog 48 h after each of three sand fly exposures. The induration score is an arbitrary scale corresponding to the area of induration and redness resulting from multiple bites where 1≤1 cm^2^, 2≤5 cm^2^, 3≤10 cm^2^ and 4>10 cm^2^. (C) Representative H&E staining of biopsies taken from sand fly bite sites prior to exposure (E0) and 48 h after each of three sand fly exposures (E1–E3). Note marked cellular infiltrate within dermis and thickening of epidermis in E3. (D) Immunohistochemical labeling of tissue sections from E3 demonstrating the presence of abundant CD3+T cells (CD3), macrophages (Mac387) and eosinophil granules (Luna stain).

In rodent models, cellular immunity characterized by a Th1 delayed type hypersensitivity (DTH) response to sand fly salivary proteins protected animals from cutaneous and visceral leishmaniases [16],[17]. Up to date, there is no information pertaining to the presence and nature of cellular immunity to sand fly saliva in dogs, the main reservoirs of ZVL [5]. Here, we explored the early kinetics of anti-saliva immunity in dogs following exposure to bites of *Lu. longipalpis*, the vector of *L. i. chagasi* in Latin America. Seven of nine beagles showed specific anti-saliva antibodies one week after the third exposure to sand fly bites (Figure 1A). Apart from a single dog with a mixed IgG_2_/IgG_1_ antibody response, these animals showed a strong IgG_2_ response and no IgG_1_ (Figure 1A). To investigate whether dogs exposed to sand fly bites develop a DTH response, we measured the skin induration at the bite site following each sand fly exposure. Following the second exposure, a small induration was observed in the 7 dogs that produced significant levels of antibodies (Figure 1B). This was characterized by a localized erythema, swelling and thickening of the skin. The intensity and duration of the observed induration was significantly increased following the third sand fly exposure lasting up to 96 h following sand fly bites (Figure 1B). No reaction was observed in naive animals after the first exposure. Histological analysis of the induration site 48 h following the first and second exposure shows minimal inflammation characterized by scattered perivascular lymphocytes and rare neutrophils within the superficial dermis (Figure 1C). A dramatic increase in the cellular infiltrate was noted 48 h following the third exposure, characterized by a prominent thickening of the epidermis and multifocal infiltrates of lymphocytes, macrophages and eosinophils (Figure 1D). The timing of the reaction as well as the nature of the infiltrate established that sand fly saliva induces a DTH reaction in the skin of dogs after repeated exposures.

### Salivary proteins LJL143 and LJM17 produce a DTH response in dogs

**Figure 2:**
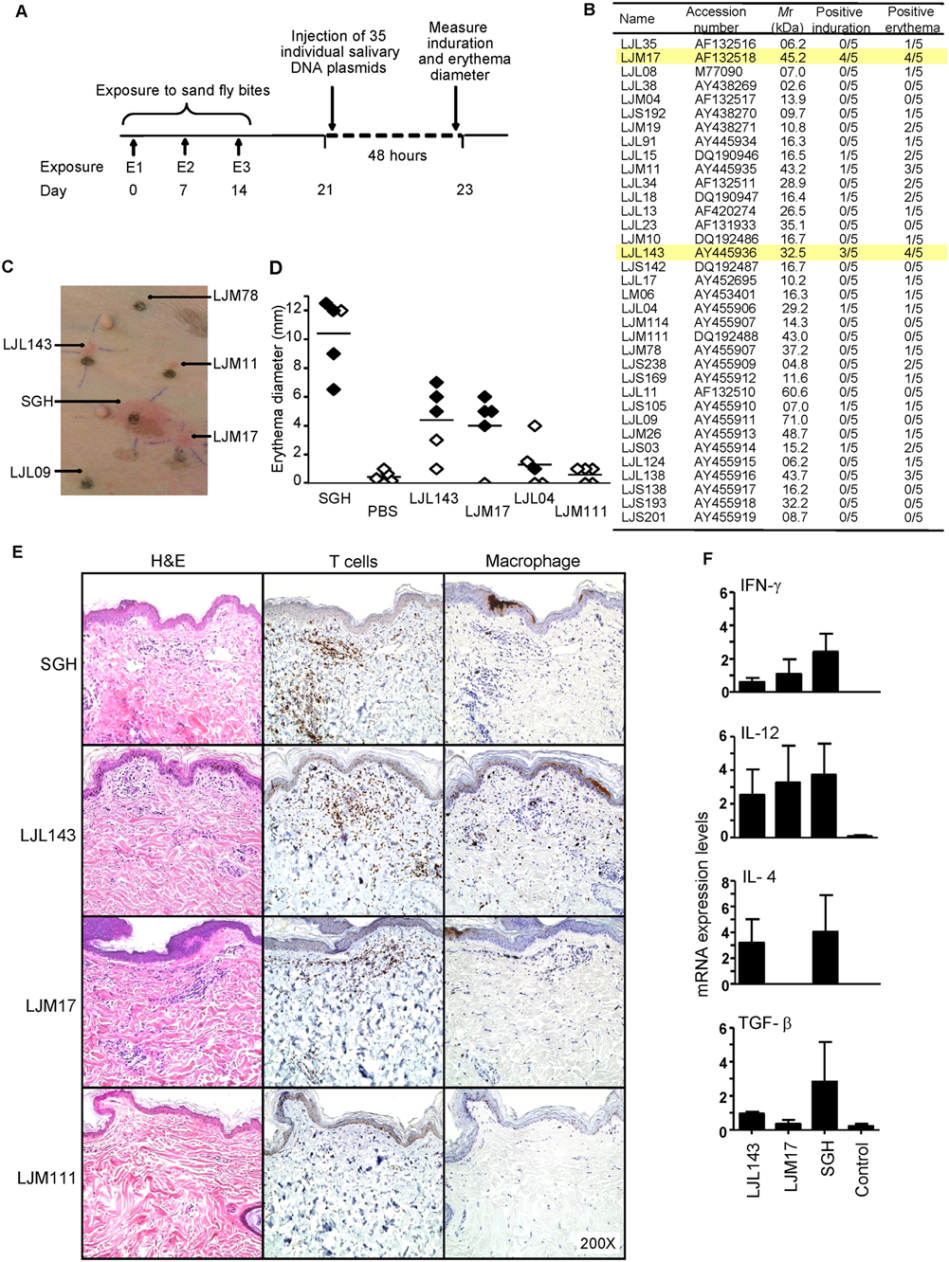
Identification of salivary proteins from *Lu. longipalpis* that produce a cellular immune response in dogs. (A) A schematic representation of the reverse antigen screening approach based on the intradermal injection of DNA plasmids in dogs previously exposed to sand fly bites (first exposure, E1; second exposure, E2; third exposure, E3). (B–F) Dogs pre-exposed to sand fly bites were challenged intradermally with DNA plasmids and one pair of salivary gland homogenate (SGH) and PBS (positive and negative controls, respectively) and investigated 48 h post-injection. (B) The number of dogs showing local induration and/or erythema at the site of injection for 35 DNA plasmids coding for secreted salivary molecules. Yellow bars highlight the response of dogs to LJM17 and LJL143. (C) Photograph to demonstrate specificity of the cellular reaction to DNA plasmids and SGH. (D) The diameter of erythema in the absence (◊) or presence (♦) of induration for each dog at the site of injection of SGH, PBS, LJL143 and LJM17 (reactive plasmids) and LJL04 and LJM111 (intermediate and non-reactive plasmids, respectively). (E–F) Skin biopsies (6mm) obtained from injection sites were cut in half and processed for histology and RNA extraction. (E) Representative H&E staining and immunohistochemical labeling of dermal T cells (anti-CD3) and macrophages (Mac387) at the injection sites of SGH, LJL143, LJM17 and LJM111. Note marked dermal infiltrates of inflammatory cells characterized as CD3+ T cells and scattered macrophages (Mac387) in the SGH, LJL143 and LJM17. There is no inflammation with LJM111. (F) Reverse-transcriptase quantitative PCR showing the expression levels of IFN-γ, IL-12, IL-4 and TGF-β for LJL143, LJM17, a pair of SGH and control (a mix of PBS and empty plasmid) 48 h post-injection. Error bars represent means±S.E.

**Figure 3:**
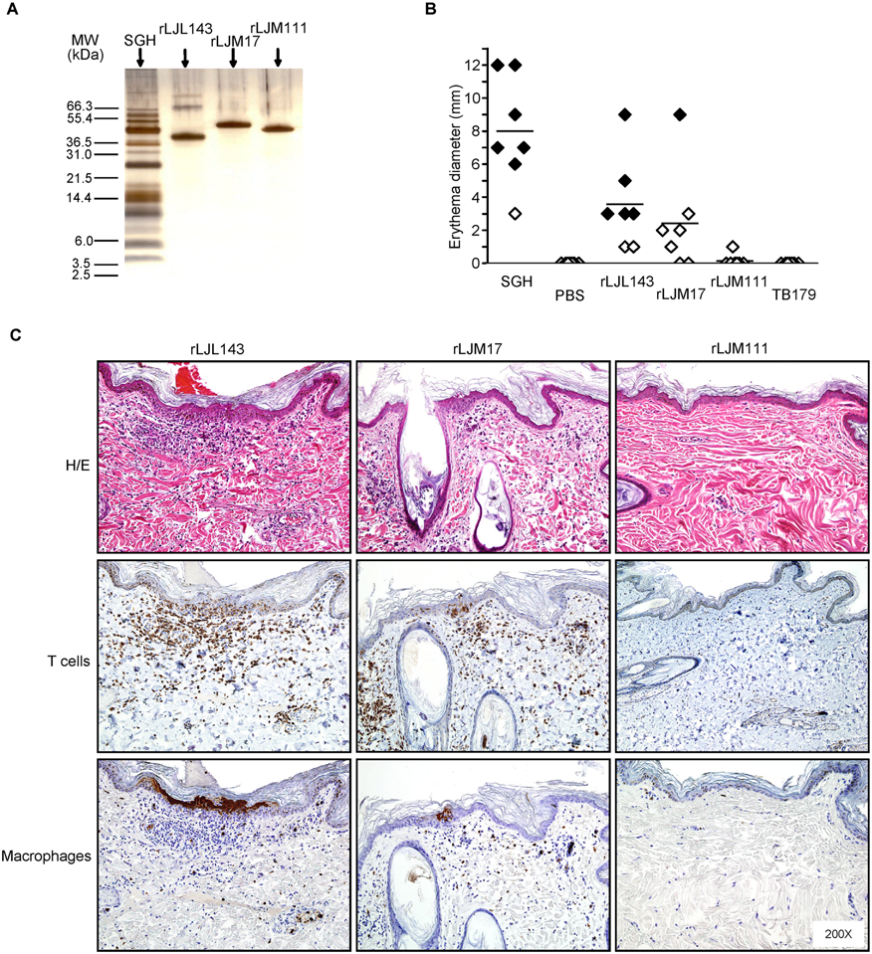
Sand fly salivary recombinant proteins produce a DTH response in dogs previously exposed to sand flies. (A) Purity of the recombinant salivary proteins produced by HEK-293F mammalian cells and purified by a HPLC nickel trap column. (B) The diameter of erythema in the absence (◊) or presence (♦) of induration for each dog at the site of injection 48 h after challenge with salivary gland homogenate (SGH), PBS, recombinant proteins rLJL143 and rLJM17 (reactive), rLJM111 (non-reactive) and a non-related tick recombinant protein TB179. (C) Representative H&E staining and immunohistochemical labeling of T cells (anti-CD3) and macrophages (Mac387) at the injection sites of rLJL143, rLJM17 and rLJM111. Note marked dermal infiltrates of inflammatory cells characterized as CD3^+^ T cells and scattered macrophages (Mac387) with rLJL143 and rLJM17; rLJM111 is negative.

Immunization with a single DTH inducing salivary molecule conferred protection from cutaneous and visceral leishmaniases in rodent models [16],[17]. To identify the salivary molecules responsible for the generation of a DTH response in dogs, we screened 35 DNA plasmids encoding secreted salivary proteins of *Lu. longipalpis* [18] using a novel approach we termed reverse antigen screening (RAS). Five dogs were exposed to sand fly bites then injected individually with up to 38 samples including three controls (Figure 2A). Out of the 35 salivary DNA plasmids only four (LJL143, LJM17, LJM11 and LJL138) induced a macroscopic DTH response 48 h after challenge defined by a strong erythema with or without palpable induration in at least three of five dogs (Figure 2B). This DTH response was highly specific as shown in Figure 2C. Since induration is an important indicator of cellular recruitment, we focused on LJL143 and LJM17 that produced the strongest combined responses in at least 3 dogs (Figure 2B,D). Histological analysis of injection sites 48 h post-challenge showed that LJL143 and LJM17 induce a typical DTH response characterized by considerable lymphocytic infiltration with few macrophages (Figure 2E). This recruitment was comparable to that of SGH (positive control) and was absent for LJM111 (negative control) as well as the vector control and PBS (data not shown). Analysis of the DTH site for expression of selected cytokines associated with Th1 or Th2 responses showed an appreciable induction of IL-12, a moderate expression of IFN-γ and low expression of TGFβ for LJM17 (Figure 2F). LJL143 showed a mixed response with IL-12 and IL-4 expression (Figure 2F). In comparison, SGH showed considerable expression levels of the four investigated cytokines (Figure 2F). There was minimal to no expression of any of the cytokines tested in negative controls (Figure 2F). To validate the specificity of the observed antigenic properties of LJL143 and LJM17 plasmids, 300 ng of purified recombinant proteins (Figure 3A) were injected in the five dogs previously exposed to DNA plasmids and in two more dogs pre-exposed to sand fly bites alone. In addition, 300 ng of rLJM111, a non-reactive sand fly salivary molecule, 300 ng of rTB179, a non-related tick salivary protein (negative controls) and a pair of SGH (positive control) were simultaneously injected. A clear DTH response was observed 48 h following the injection of rLJL143 and rLJM17. The DTH response was characterized by erythema with or without palpable induration (Figure 3B) and cellular infiltration (Figure 3C) comparable to those observed following the injection of DNA plasmids (Figure 2C,E). As predicted, rLJM111, rTB179 and PBS showed no erythema or induration (Figure 3B). The absence of cellular infiltration was confirmed by histology for rLJM111 (Figure 3C). It is worth noting that LJM111 remained non-reactive following the injection of the recombinant protein despite the fact that five dogs were pre-challenged with the DNA plasmid encoding that protein.

### Strong induction of a Th1 humoral and cellular immunity in dogs immunized with LJL143 and LJM17

**Figure 4:**
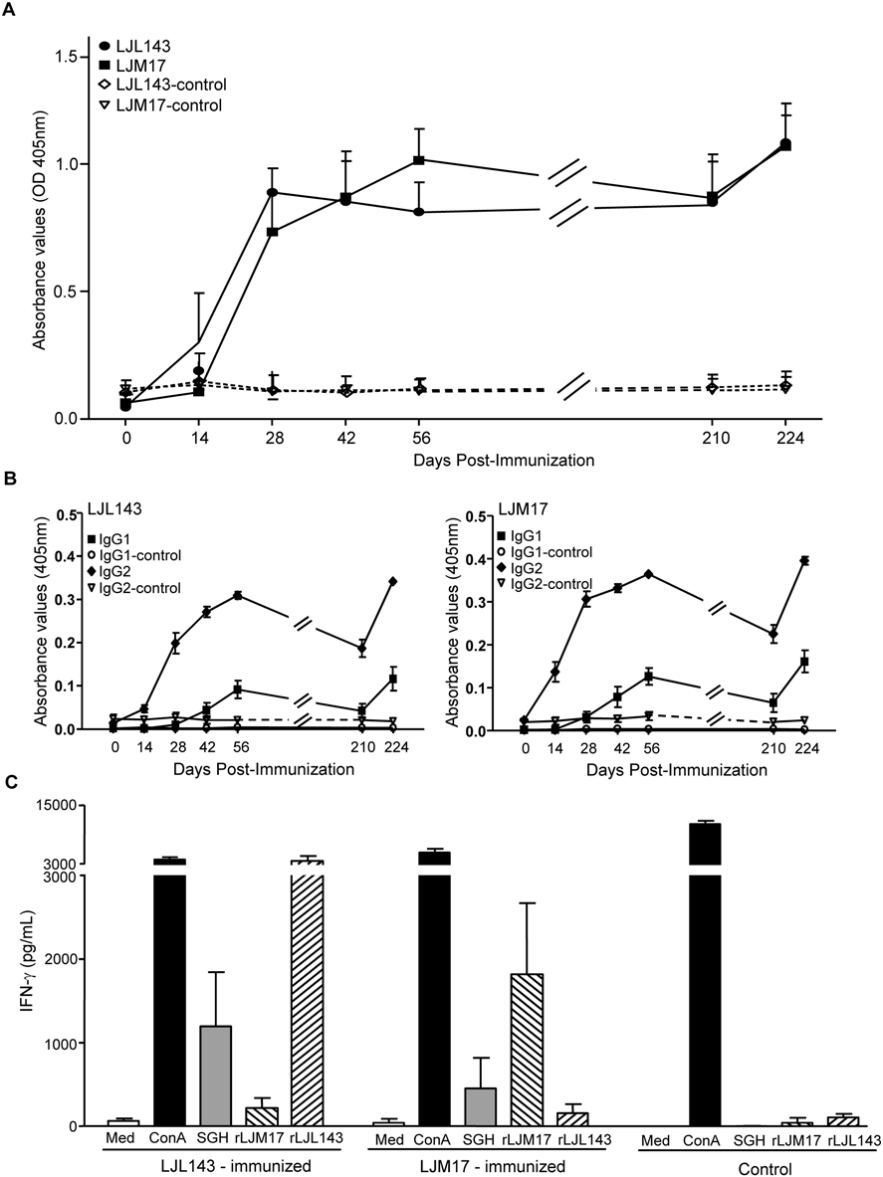
Dogs immunized with the *Lu. longipalpis* salivary molecules LJM17 or LJL143 develop strong and specific humoral and cellular immune responses. (A) Total IgG and (B) IgG_1_ and IgG_2_ antibody levels up to day 224 in dogs immunized with either LJM17 (n = 5), LJL143 (n = 5) or the empty plasmid (n = 5). LJL143- and LJM17-immunized dogs were tested using the appropriate recombinant proteins (LJL143 and LJM17). Dogs immunized with the empty vector (control dogs) were tested against both recombinant proteins for IgG, (LJL143-control, LJM17-control), IgG_1_ (IgG_1_-control) and IgG_2_ (IgG_2_-control). (C) *In vitro* IFN-γ production by PBMC from LJL143- and LJM17-immunized and control dogs stimulated with media (Med), ConcavalinA (ConA), salivary gland homogenate (SGH), rLJM17 or rLJL143 two weeks after the final vaccination. Error bars represent means±S.E.

It is well established that a Th1 cell-mediated immunity (CMI), characterized by the production of IFN-γ, is critical for protection from *Leishmania* infection [19]. Using RAS, LJL143 and LJM17 were identified as vaccine candidates following the induction of a DTH response in dogs previously exposed to *Lu. longipalpis* bites (Figures 2 and 3). Subsequently, naïve dogs were immunized with LJL143 and LJM17 using DNA plasmids followed by a recombinant protein boost. Both LJL143 and LJM17 induced a strong humoral response (Figure 4A) that was efficiently recalled by a viral vector boost (Figure 4A,B). Furthermore, IgG_2_ was the predominant IgG subclass in immunized dogs (Figure 4B). Following the viral vector boost, PBMC from control or LJL143- and LJM17-immunized dogs were isolated and stimulated with recombinant proteins or SGH. PBMC from LJL143-immunized dogs produced over 3600 pg/ml and 1200 pg/ml of IFN-γ following stimulation with rLJL143 and SGH, respectively (Figure 4C). In LJM17-immunized dogs, IFN-γ production was also high at 1813 pg/ml and 446 pg/ml after stimulation with rLJM17 and SGH, respectively (Figure 4C), Moreover, IFN-γ production was specific to the recombinant proteins since stimulation of PBMC from LJL143-immunized dogs with rLJM17 produced background levels of IFN-γ and vice versa (Figure 4C).

### Bites of *Lutzomyia longipalpis* sand flies induce a strong focal and systemic adaptive immune response in dogs immunized with LJL143 or LJM17

**Figure 5:**
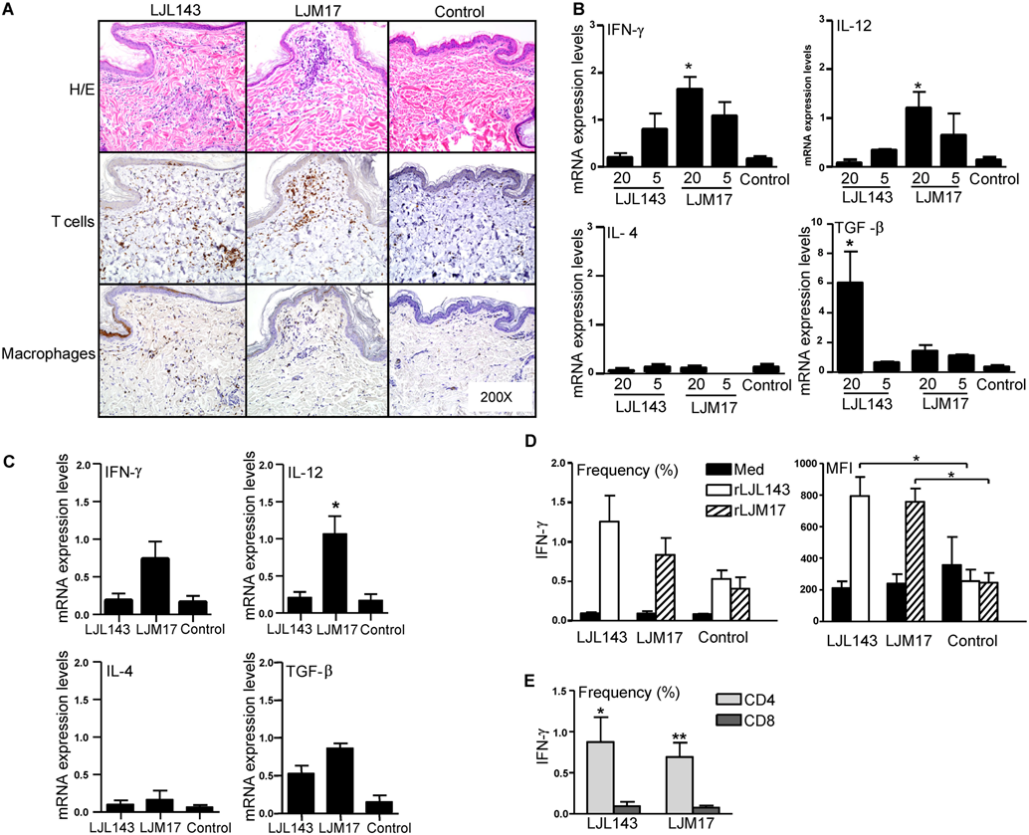
Bites of *Lu. longipalpis* sand flies induce a strong focal and systemic adaptive cellular immune response in dogs immunized with LJL143 or LJM17. (A–C) Dogs were exposed to uninfected and infected sand flies for 10 min one month after the final immunization with either LJM17, LJL143 or the empty plasmid (control). (A–C) Skin biopsies (6mm) obtained from bite sites 48 h post challenge with 20 and five uninfected and 10 infected sand flies were cut in half and processed for histology and RNA extraction. (A) Representative H&E staining and immunohistochemical labeling of T cells (anti-CD3) and macrophages (Mac387) at the bite sites of 20 uninfected sand flies in LJL143- and LJM17-immunized and control dogs. (B) Reverse-transcriptase quantitative PCR showing the expression levels of IFN-γ, IL-12, IL-4 and TGF-β at the bite sites of 20 or five uninfected sand flies in LJL143- and LJM17-immunized and control dogs (for control dogs RNA was combined from sites of 20 and 5 uninfected sand fly bites). (C) Same as (B) using 10 infected sand flies. Histological sections from bite sites of five uninfected and 10 infected sand flies are provided as Figure S1 and Figure S2, respectively. (D–E) PBMC from LJL143- and LJM17-immunized and control dogs obtained one week after exposure to sand flies. (D) Frequency and mean fluorescence intensity (MFI) of CD3^+^ T cells following stimulation with medium, rLJL143 or rLJM17. (E) Frequency of CD4^+^ and CD8^+^ T cells expressing IFN-γ in PBMC from LJL143- and LJM17-immunized dogs. Error bars represent means±S.E. * *P*<0.05, ** *P*<0.01.

LJL143- and LJM17-immunized dogs produced a strong Th1 systemic humoral and cellular response to the corresponding salivary proteins (Figure 4). To determine whether this immunity is maintained under natural conditions, these dogs were exposed to sand fly bites, the natural route of transmission. A distinct focal cellular infiltration of CD3^+^ cells and a few scattered macrophages was observed 48 h following the bites from 20 or five uninfected flies in dogs immunized with LJM17 or LJL143 (Figure 5A, Figure S1). RNA from biopsies taken at the bite site was used to determine the expression of key cytokines 48 h after sand fly bites. Following bites by 20 uninfected flies, LJM17-immunized dogs showed a polarized Th1 immune response characterized by a significant induction of IFN-γ and IL-12 (*P*<0.03) with low levels of IL-4 and the regulatory cytokine TGF-β (Figure 5B). This expression profile was also observed in response to 5 uninfected sand fly bites. Interestingly, LJL143-immunized dogs induced a different profile when challenged with 20 compared to 5 sand flies. TGF-β was the dominant cytokine induced following 20 bites (*P*<0.03) with low expression levels for IFN-γ, IL-12 and IL-4 (Figure 5B). In contrast, LJL143-immunized dogs challenged with 5 sand flies produced five times the expression levels of IFN-γ compared to those observed in controls and low levels of IL-4 expression (Figure 5B). To assess whether altered feeding behavior of infected sand flies (caused by parasite blockage of the stomodeal valve) influences the nature of the recall response, LJL143- and LJM17-immunized dogs were simultaneously exposed to the bites of 10 sand flies infected with *L. i. chagasi*. Forty-eight h following challenge with infected sand flies, both groups of dogs produced a strong focal cellular infiltration (Figure S2) and a cytokine profile similar to that of uninfected sand flies (Figure 5C). Analysis of PBMC one week after sand fly challenge showed that the frequency of CD3^+^ cells producing IFN-γ following stimulation with the appropriate recombinant proteins was considerably larger in LJL143- and LJM17-immunized dogs and showed a significantly higher mean fluorescence intensity (MFI) (*P*<0.05) compared to cells from control dogs (Figure 5D). Further analysis showed that CD3^+^ CD4^+^ T cells were the source of IFN-γ in immunized dogs (Figure 5E).

### Macrophages efficiently kill *Leishmania infantum chagasi in vitro* following the addition of SGH-stimulated LJL143- and LJM17-specific lymphocytes

**Figure 6:**
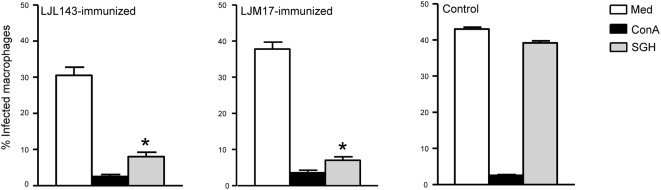
Macrophages efficiently kill *L. i. chagasi in vitro* following the addition of autologous lymphocytes from LJL143- and LJM17-immunized dogs stimulated with SGH. Percent of infected macrophages 72 h after the addition of autologous lymphocytes alone (Med), or together with ConA or SGH from PBMC of LJL143- and LJM17-immunized and control dogs. The percentage of amastigote-infected macrophages was evaluated by microscopic examination of Giemsa-stained preparations. Error bars represent means±S.E. * *P*<0.0001.

Immunization of dogs with the salivary proteins LJL143 and LJM17 resulted in a strong focal and systemic CMI against sand fly bites, the natural route of *Leishmania* transmission. To test whether this immunity has an adverse effect on *Leishmania* parasites, macrophages from PBMC of LJL143- and LJM17-immunized or control dogs were infected with *L. i. chagasi in vitro*. The addition of SGH-stimulated autologous lymphocytes from LJL143- and LJM17-immunized dogs resulted in a 74% and 82% (*P*<0.0001) reduction of infection in macrophages, respectively (Figure 6). In contrast, the percent of infected macrophages was not altered by the addition of SGH-stimulated autologous lymphocytes from PBMC of control dogs.

## Discussion

We propose the inclusion of salivary antigens of the sand fly *Lu. longipalpis*, the only established vector of *L. i. chagasi* in Latin America, as a component of anti-*Leishmania* vaccines against CVL. This is based on the 1) induction of a strong Th1 cellular immune response, the hallmark of protection against leishmaniasis, in dogs immunized with two novel salivary proteins from the vector *Lu. longipalpis*; 2) efficient recall of this Th1 immunity in the skin at the bite site of infected sand flies, important when considering that *Leishmania* are co-deposited with salivary proteins during probing and feeding; 3) evidence that immunity to these salivary proteins has an adverse effect on *L. i. chagasi*.

From a repertoire of 35 salivary molecules from *Lu. longipalpis*, RAS correctly identified two salivary proteins, LJL143 and LJM17, as inducers of CMI in dogs. It is important to note that the antigens identified in this study differ from those eliciting immune responses in rodent models [15],[16],[17]. LJM19, a salivary molecule from *Lu. longipalpis*, conferred protection from visceral leishmaniasis through induction of a strong DTH response in hamsters [16] but induced a weak response in dogs (Figure 2). This may be due to the restriction imposed by the repertoire of the major histocompatibility complex class II molecules present in different animals. Therefore, one can expect that immunogenic antigens will vary in different animals. This demonstrates the power of RAS in large laboratory animals such as dogs for the rapid screening of antigens inducing CMI. For this reason, the RAS technique represents a significant improvement in the selection of appropriate vaccine candidates whereby it permits screening of populations targeted by a vaccine, including dogs and humans, for antigens inducing a cellular response.

In dogs pre-exposed to sand fly bites, LJL143 and LJM17 induced a distinct cellular infiltration characterized by CD3^+^ lymphocytes, macrophages and notably, the absence of eosinophils. This differs from the response to natural bites that produced a mixed response including a substantial number of eosinophils and suggests that LJL143 and LJM17 are not likely to induce an allergic response typically associated with exposure to insect saliva. This was further supported by the lack of an allergic response in immunized dogs following challenge by up to 35 sand fly bites, an important consideration in the selection of salivary vaccine candidates.

Dogs immunized with LJL143 or LJM17 showed a consistent systemic adaptive immune response indicative of a Th1 profile. This was demonstrated by the dominance of IgG2 antibodies throughout the study period and the substantial production of IFN-γ by CD3^+^CD4^+^ T cells stimulated with SGH or recombinant proteins. Considering that beagles are out bred, this consistency is encouraging and bodes well for the use of these antigens in the field.

A *Leishmania* vaccine has a better chance of success under field conditions if it generates a rapid immune response in the skin following the deposition of a relatively low dose of parasites [20] by an infective sand fly. This immune response should be specific to an antigen delivered during the bite, be it *Leishmania* antigens or salivary proteins that are co-injected into the bite site. Sand fly bites, uninfected and infected, elicited a distinct and comparable cellular recruitment mediated by lymphocytes at the bite site in dogs immunized with either LJL143 or LJM17. The cytokine profile, assessed 48 h post bites, was characterized by the presence of IFN-γ and IL-12 and the absence of IL-4 in LJM17-immunized dogs challenged with 5, 10 or 20 sand fly bites. This profile was similar in LJL143-immunized dogs challenged with 5 sand flies. However, the response in these dogs to 10 and 20 bites was low with the exception of TGFβ. The presence of high levels of TGFβ, a regulatory cytokine, suggests that this may be a regulatory mechanism to dampen an earlier burst of IFN-γ production. Thus, the differences observed in cytokine levels may be explained by different kinetics of the immune response to the two molecules combined with the different number of bites received. Indeed, PBMC of LJL143-immunized dogs produced high levels of IFN-γ following stimulation with SGH.

We hypothesized that anti-saliva immunity if generated against a Th1 polarizing antigen can potentially have an adverse effect on the parasites deposited together with saliva. *In vitro*, macrophages infected with *L. i. chagasi* efficiently killed the parasites following the addition of autologous T cells from LJL143- and LJM17-immunized dogs stimulated by SGH showing a 74% and 82% reduction of infection in macrophages respectively. This demonstrates a clear effect of anti-saliva immunity on *Leishmania* parasites. How this anti-saliva immunity plays out *in vivo* remains to be fully elucidated. It could act through an initial indirect killing of *Leishmania in situ*, acceleration of specific anti-*Leishmania* immunity or a combination of both [14],[15],[16],[17]. Acceleration of anti-*Leishmania* immunity can occur as a result of a more rapid processing of killed parasites or through the effect of an altered cytokine milieu on the nature and commitment of cells recruited to the site by anti-saliva immunity.

In conclusion, induction of immune correlates of protection in dogs immunized with salivary proteins from *Lu. longipalpis* is a strong predictor that these molecules will be an advantageous addition to an anti-*Leishmania* canine vaccine. Sand fly salivary molecules have been neglected as a component of anti-*Leishmania* vaccines despite their reported immunogenicity in rodent models and humans [16],[17],[21],[22], and their unique advantage as a permanent feature of natural transmission. Salivary proteins can provide a novel source of antigens that may complement or synergize promising *Leishmania*-based vaccines providing an independent arm of the immune response that could be of value in the control of leishmaniasis.

## Materials and Methods

### Animals

One to two year old female beagles (Marshall Farms) were housed at the NIH animal facility following the Animal Care and User Committee guidelines. Four to seven day old *Lutzomyia longipalpis* female sand flies (Jacobina colony) were used in experiments. Salivary glands were sonicated, centrifuged at 10,000 g for 3 min and used immediately. *L. i. chagasi* (BA262 strain) promastigotes were cultured as previously described [16].

### DNA plasmids

DNA plasmids were constructed and purified as previously described [16], filter sterilized and stored at −70°C.

### Recombinant proteins

Specific *Lu. longipalpis* salivary cDNA containing a histidine tag at the 3′ end were cloned into the VR2001-TOPO expression vector [23]. HEK-293F cells were transfected and supernatants collected at 72 h. Expressed proteins were purified by HPLC (DIONEX) using a HITRAP Chelating HP column (GE HealthCare) charged with Ni_2_SO_4_ 0.1M. Proteins were eluted using an imidazole gradient, dialyzed against PBS and stored at −70°C.

### Recombinant canarypoxvirus

Canarypoxviruses from ALVAC vectors expressing the LJL143 (vCP2389) or LJM17 (vCP2390) were generated as previously described [24]. PUREVAX ferret distemper vaccine (Merial) was used as control.

### Sand fly infection

Sand flies were fed through a chick skin membrane on a suspension of 3×10^6^ *L. i. chagasi* procyclic promastigotes/ml of heparinized blood containing penicillin and streptomycin. Flies with mature infections were used for transmission.

### Exposure of dogs to sand fly bites

Dogs were sensitized three times weekly with 20 sand flies placed in custom made chambers and secured to the shaved side of the neck with a Velcro collar for 10 min. For assessing the skin immune response, five and 20 uninfected and 10 infected sand flies were placed in small vials and hand-held to marked sites on the shaved belly of dogs for 10 min. Dogs were handled without any chemical restraint.

### DTH measurement

The diameter of erythema and the induration (elevation over 1 mm) on the skin of dogs were measured 48 h post-injection.

### Skin biopsies

6mm skin punch biopsies (Acuderm) were cut in two. One half was fixed in neutral-buffered formaldehyde (10% formalin) for histology and the other was stored in RNALATER (Sigma-Aldrich) for RNA extraction.

### Histology and Immunohistochemistry

Formalin fixed skin biopsies were embedded in paraffin. Four µm sections were processed for staining with hematoxylin and eosin (H & E) and Luna's stain. Additional sections were labeled with anti-CD3 and Mac387. Sections were incubated with primary rabbit anti-human CD3 (Dako, Glostrup, Denmark) and mouse anti-human Mac387 (Serotec, Raleigh, NC) at 1∶300 and 1∶400 respectively for 1 h. For CD3, a secondary biotinylated goat anti-rabbit antibody was used at 1∶500 for 15 min (Vector Laboratories, Burlingame, CA) and detected by R.T.U. VECTASTIN Elite ABC reagent (Vector) and DAB chromagen. For Mac387, a secondary antibody labeled with Mach 4 HRP Polymer (Biocare Medical, Concord, CA) was used following the manufacturer's recommendation and detected by DAB chromagen.

### Reverse antigen-screening (RAS)

Dogs pre-exposed to sand fly bites were anesthetized and randomly injected intradermally with 40 µL of 35 salivary DNA plasmids (20 ug each) or recombinant proteins (300 ng) diluted in PBS and separated from each other by ∼15mm. Controls included PBS, a pair of *Lu. longipalpis* SGH (1 µg), 20 µg of control vector or 300 ng of rTB179, a tick recombinant salivary protein.

### Immunization of dogs

At day 0, five dogs per group were immunized intradermally (ID) in the ear pinna with 500 µg of LJL143 DNA plasmid (group one), LJM17 DNA plasmid (group two) and VR2001 control vector (group three). The dogs were given a second and third immunization at days 14 and 28 with 500 µg of the appropriate DNA plasmids injected in both thighs intramuscularly (IM) coupled to electroporation (Sphergen). At day 42, the dogs were immunized ID with 100 µg of rLJL143 for group 1, rLJM17 for group 2 or BSA for group 3 together with 300 µg CpG ODN in 20% EMULSIGEN (MVP laboratories). At day 210, the dogs received a vaccine booster (IM) in the left quadriceps using 10^8^ pfu of recombinant canarypoxvirus expressing LJL143 or LJM17 for group two, and PUREVAX control canarypoxvirus for group three.

### Direct enzyme-linked immunosorbent assay (ELISA)

Microtiter plates (MAXSORP, Nunc) were coated with 100 µl of 2 µg/ml rLJM17 or rLJL143 or *Lu. longipalpis* SGH (five salivary gland pairs/ml) overnight at 4°C. Plates were blocked with 4% fetal bovine serum (FBS) in PBS-TWEEN 0.05% at RT for 2 h. A100 µl of dog sera (1∶50) was incubated for 1 h at 37°C. After three washes with PBS-T, sheep anti-dog IgG (1∶5000), goat anti-dog IgG_1_ (1∶500) or sheep anti-dog IgG_2_ (1∶500) phosphatase alkaline-conjugated antibodies **(**Bethyl Laboratories Inc.) were incubated for 1 h at 37°C. Plates were developed with *p*-nitrophenylphosphate (Sigma-Aldrich) and absorbance was read at 405nm using a SPECTRAMAX Plus (Molecular Devices).

### Capture ELISA

PBMC were isolated as previously described [25]. A million cells per well of a 96 well-plate (Research & Diagnostic systems) were cultured for 72 h in 500 µl of RPMI supplemented with 20% heat-inactivated FBS, 2mM L-glutamine, 100 units/ml penicillin and 100 µl/ml streptomycin (cRPMI) with either two pairs of SGH, ConA (4 µg), rLJM17 (4 µg) or rLJL143 (4 µg). IFN-γ production was measured from supernatants using QUANTIKINE ELISA (Research & Diagnostic Systems). Absorbance (405 nm) was measured using SPECTRAMAX Plus (Molecular Devices).

### Real-time PCR

Isolation of RNA from skin and first strand cDNA synthesis was performed as previously described [16]. DNA was amplified with specific dog primers (Operon Biotechnologies, Inc.) and probes (Roche Diagnostics) for IFN-γ, IL-12, IL-4 and TGF-β as previously described [16]. The expression level of genes of interest was normalized to GAPDH levels.

### Flow cytometry

Two million PBMC were cultured in a ml of cRPMI for 18 h in the presence of either ConA (4 µg), rLJM17 (20 µg) or rLJL143 (20 µg) at 37°C in 5% CO_2._ Cells were incubated with 2 µM final concentration of GolgiStop (BD Pharmingen) for 4 h, washed with PBS-5% FBS, and blocked with PBS-10% FBS for 30 min at 4°C. Cells were stained with FITC-labeled anti-CD3 (CA17.2A12, BD Pharmingen) and ALEXA FLUOR 647-labeled anti-CD8 (YCATE55.9, BD Pharmingen) for 30 min at 4°C, washed twice, fixed and permeabilized with CYTOFIX/CYTOPERM Plus (BD Pharmingen) and stained with PE-labeled anti-IFN-γ (CC302, BD Pharmingen). A minimum of 200,000 cells were acquired using a FACSCalibur flow cytometer (BD Biosciences) and analyzed with CELLQUEST Pro software.

### *In vitro Leishmania* killing assay

Canine monocyte-derived macrophages were prepared as previously described [26]. PBMC collected from immunized dogs were plated in 8 well chamber slides (BD FALCON) at 5×10^6^ cells per ml and incubated for 30 min at 37°C with 5% CO2. Non-adherent cells (autologous T cells) were removed and cultured separately. After 5 d of culture, macrophages were infected with stationary phase *L. i. chagasi* at a 5∶1 parasites to macrophage ratio for 2 h at 37°C- 5% CO_2_. Non-internalized parasites were removed by gentle washing. Infected macrophages were cultured for 72 h in the presence of autologous lymphocytes at a 2∶1 lymphocyte to macrophage ratio and stimulated with *Lu. longipalpis* SGH (2 pairs) or ConA (4 µg). The percentage of infected macrophages was assessed by microscopic examination of Giemsa-stained preparations.

### Statistical analysis

Statistical significance was tested with the two-tailed student's *t*-test using Graph Pad 4.0 Prism Software.

## Supporting Information

Figure S1 Bites of *Lu. longipalpis* sand flies induce a strong focal cellular immune response in dogs immunized with LJL143 or LJM17. Dogs were exposed to 5 uninfected sand flies for 10 min one month after the final immunization with either LJM17, LJL143 or empty plasmid (control). Skin biopsies (6mm) obtained from bite sites 48 h post challenge were processed for histology. Representative H&E staining and immunohistochemical labeling of T cells (anti-CD3) and macrophages (Mac387) at the bite sites in LJL143- and LJM17-immunized and control dogs.
(10.09 MB TIF)

Figure S2 Bites of *L. i. chagasi* infected sand flies induce a strong focal cellular immune response in dogs immunized with LJL143 or LJM17. Dogs were exposed to ten *L. i. chagasi* infected sand flies for 10 min one month after the final immunization with either LJM17, LJL143 or empty plasmid (control). Skin biopsies (6mm) obtained from bite sites 48 h post challenge were processed for histology. Representative H&E staining and immunohistochemical labeling of T cells (anti-CD3) and macrophages (Mac387) at the bite sites in LJL143- and LJM17-immunized and control dogs.
(7.93 MB TIF)
