# Supplementary figures and images for: Sand Fly Salivary Proteins Induce Strong Cellular Immunity in a Natural Reservoir of Visceral Leishmaniasis with Adverse Consequences for Leishmania
Source: PLoS Pathog. 2009 May 22;5(5):e1000441. doi: 10.1371/journal.ppat.1000441 (PMC2677456; doi:10.1371/journal.ppat.1000441)

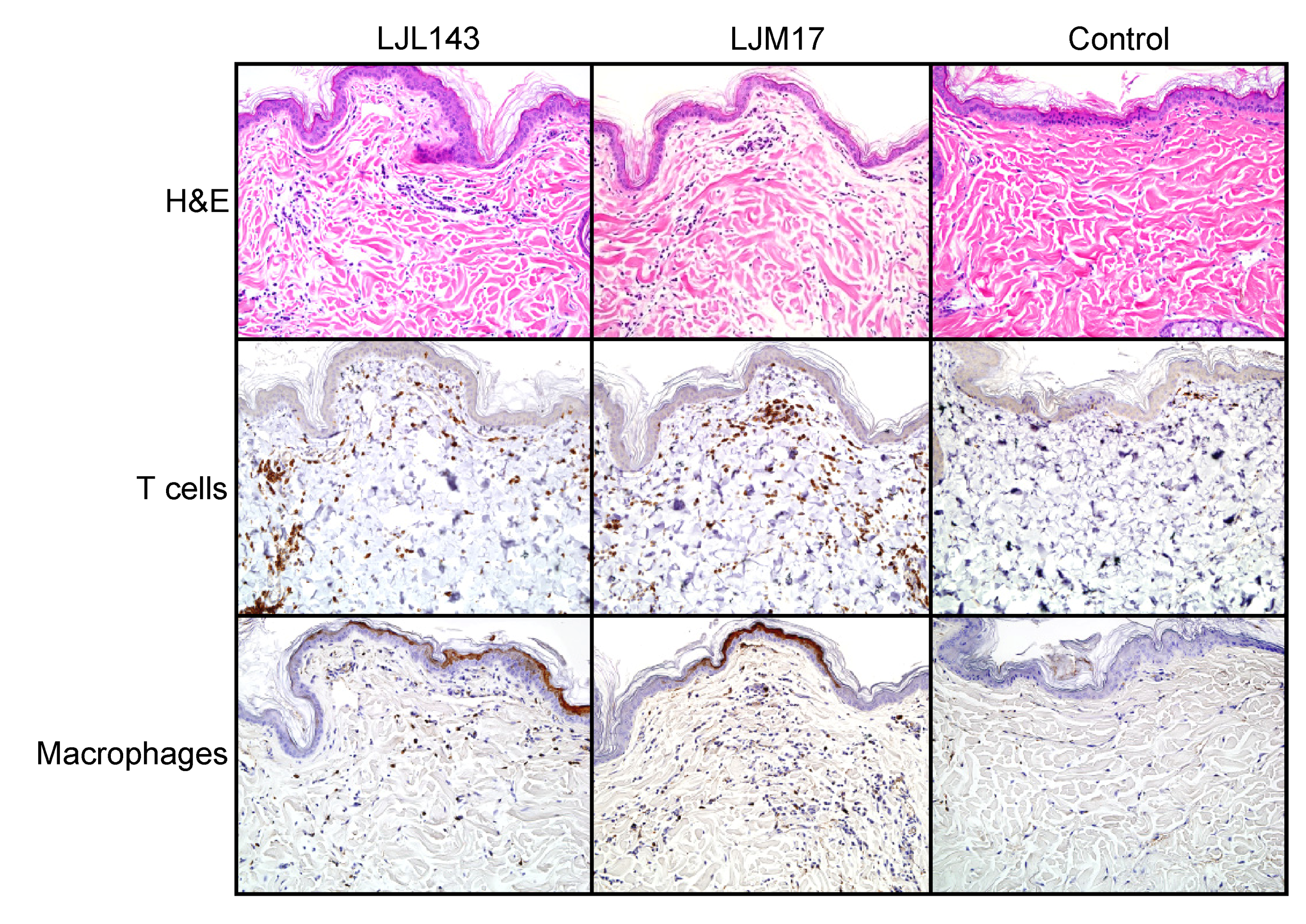

Supplement: Figure S1 — Bites of Lu. longipalpis sand flies induce a strong focal cellular immune response in dogs immunized with LJL143 or LJM17. Dogs were exposed to 5 uninfected sand flies for 10 min one month after the final immunization with either LJM17, LJL143 or empty plasmid (control). Skin biopsies (6mm) obtained from bite sites 48 h post challenge were processed for histology. Representative H&E staining and immunohistochemical labeling of T cells (anti-CD3) and macrophages (Mac387) at the bite sites in LJL143- and LJM17-immunized and control dogs. (10.09 MB TIF) [file ppat.1000441.s001.tif]

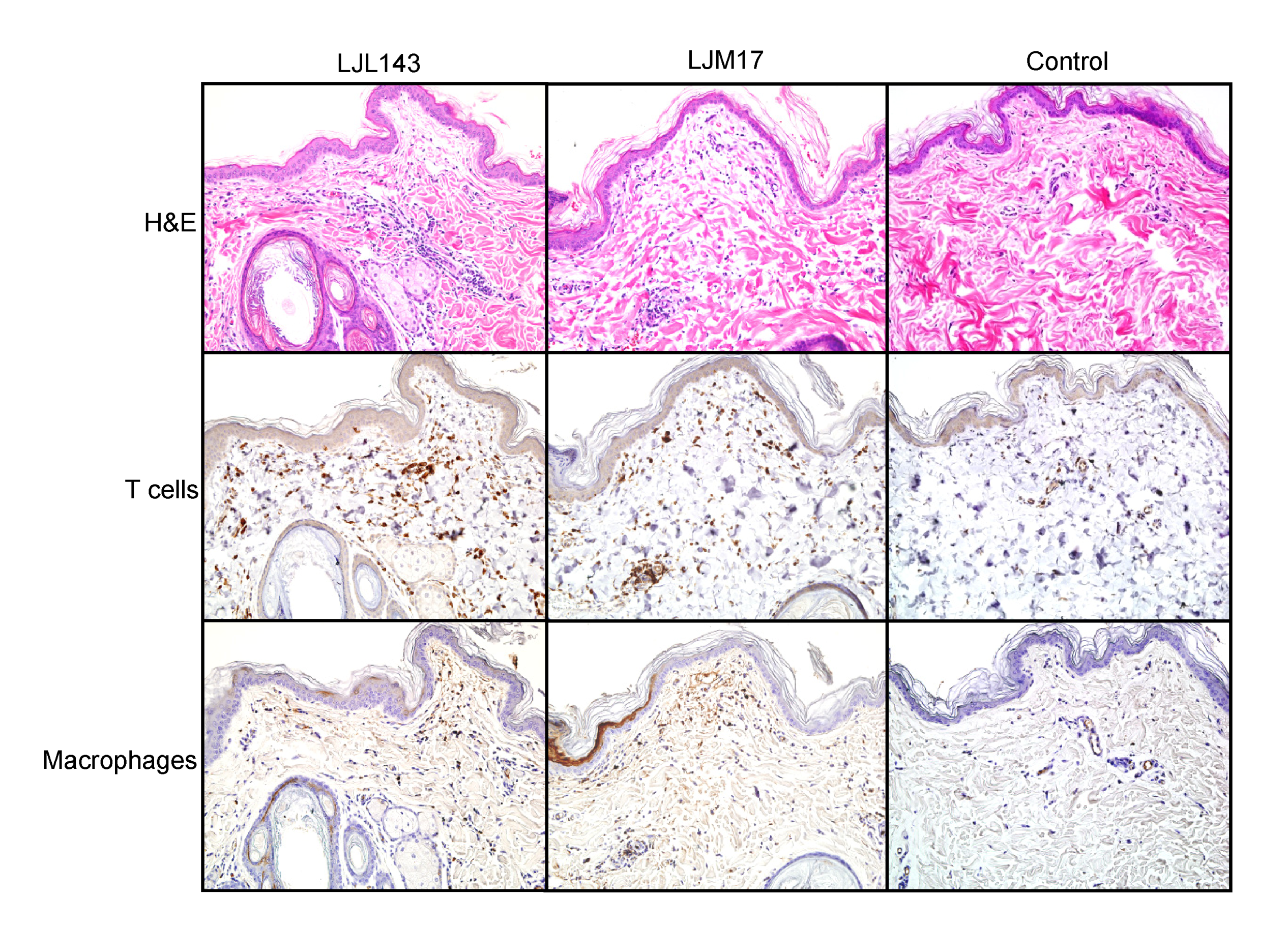

Supplement: Figure S2 — Bites of L. i. chagasi infected sand flies induce a strong focal cellular immune response in dogs immunized with LJL143 or LJM17. Dogs were exposed to ten L. i. chagasi infected sand flies for 10 min one month after the final immunization with either LJM17, LJL143 or empty plasmid (control). Skin biopsies (6mm) obtained from bite sites 48 h post challenge were processed for histology. Representative H&E staining and immunohistochemical labeling of T cells (anti-CD3) and macrophages (Mac387) at the bite sites in LJL143- and LJM17-immunized and control dogs. (7.93 MB TIF) [file ppat.1000441.s002.tif]
